# Comparison of zoonotic enteric disease outbreaks associated with ruminant and non-ruminant animal contact — United States, 2013–2021

**DOI:** 10.3389/fpubh.2026.1845080

**Published:** 2026-07-07

**Authors:** Zainab Salah, G. Sean Stapleton, Zahra Ali, Anna J. Blackstock, Megin Nichols

**Affiliations:** 1 Division of Foodborne, Waterborne, and Environmental Diseases, Centers for Disease Control and Prevention, Atlanta, GA, United States; 2 Oak Ridge Institute for Science and Education, Oak Ridge, TN, United States

**Keywords:** enteric, One Health, outbreak, ruminant, zoonotic

## Abstract

**Introduction:**

Contact with animals is a known risk factor for enteric illness outbreaks, but outbreak characteristics and pathogen distribution vary by animal type. Characterizing outbreaks associated with different animal groups can improve understanding of transmission patterns and inform prevention and response efforts.

**Methods:**

We analyzed United States outbreak data reported to CDC’s National Outbreak Reporting System from 2013 to 2021 to compare outbreaks caused by *Salmonella*, *Campylobacter*, *Cryptosporidium*, or Shiga toxin-producing *Escherichia coli* (STEC) associated with ruminants (e.g., cattle, goats, sheep) or non-ruminants (e.g., poultry, reptiles, companion animals). We compared outbreak size, demographics, healthcare use, and health outcomes.

**Results:**

Among 350 outbreaks (10,741 illnesses), 36% were associated with ruminants and 64% with non-ruminants. Ruminant-associated outbreaks were primarily caused by *Cryptosporidium* (70%) or STEC (17%), while non-ruminant outbreaks were caused by *Salmonella* (78%) or *Campylobacter* (20%). Ruminant-associated outbreaks were smaller (median: 4 vs. 14 cases) and had lower hospitalization rates (11% vs. 29%) and emergency room visit rates (14% vs. 21%). Adults aged 20–49 years accounted for a larger proportion of illnesses in ruminant-associated outbreaks, whereas non-ruminant outbreaks affected a broader age distribution, with a lower proportion of illnesses among persons aged 5–19 years.

**Discussion:**

Zoonotic enteric outbreaks differ substantially by animal contact type in pathogen distribution, outbreak size, severity, and affected populations. Ruminant-associated outbreaks disproportionately affected adults aged 20–49 years and were more frequently associated with *Cryptosporidium* or STEC, whereas non-ruminant outbreaks more commonly involved *Salmonella* and showed a more evenly distributed age pattern. These findings support the need for animal-specific risk communication, targeted prevention strategies, and routine inclusion of animal exposure questions in public health investigations to improve outbreak detection and response.

## 1 Introduction

Zoonotic transmission of enteric pathogens through direct or indirect contact with live animals is a well-established source of both sporadic illness and outbreaks in humans (1–3). Animals can harbor bacteria and parasites in their gastrointestinal tracts and shed these organisms into the environment where they may infect people through contact with feces or contaminated surfaces (2). In the United States, an estimated 450,000 enteric illnesses occur annually due to animal contact, with some illnesses resulting in severe health outcomes including septicemia, meningitis, or death (3). Recent surveillance data further highlight the public health importance of animal-associated enteric disease, with outbreaks linked to a wide range of animal species and settings (3–5).

Outbreaks of enteric illness are reported by public health agencies through the National Outbreak Reporting System (NORS), a national surveillance platform maintained by the Centers for Disease Control and Prevention (CDC) (6). NORS captures data on outbreak size, geographic location, implicated pathogens, transmission routes, and other epidemiologic characteristics (1). Within NORS, the Animal Contact Outbreak Surveillance System (ACOSS) specifically tracks outbreaks attributed to contact with animals (7). Since its implementation in 2009, ACOSS has contributed to a growing understanding of zoonotic transmission of pathogens such as non-typhoidal *Salmonella enterica*, *Campylobacter* spp., *Cryptosporidium* spp., and Shiga toxin-producing *Escherichia coli* (STEC) (5, 8–12).

Prior analyses using NORS and ACOSS data have described overall patterns in zoonotic outbreaks and pathogen-specific trends (8–12). Certain animal types have been more commonly linked to spreading specific infectious agents compared to others. For example, backyard poultry (e.g., chickens, ducks, and turkeys that are privately owned and not part of the commercial food supply) are the most common causes of animal-associated *Salmonella* infection in humans (13, 14), while ruminants (e.g., cattle, sheep, and goats) are more likely to be associated with human cryptosporidiosis outbreaks (9, 12). Ruminants are also the most frequently implicated animal category among outbreaks with a clearly attributed animal source, with approximately 75% of ruminant-associated outbreaks linked specifically to contact with cattle (5).

Despite well-known associations, important gaps remain. Most prior studies have focused on pathogen-specific outbreaks or single animal categories, and relatively few have directly compared the epidemiologic characteristics of outbreaks by type of animal contact across multiple pathogens and settings (8–12, 15–17). In particular, the distinct features of outbreaks associated with ruminant contact remain underexplored relative to those associated with non-ruminant species, limiting our understanding of how animal type may influence outbreak severity, transmission dynamics, and affected populations. Understanding these differences may enhance the effectiveness of outbreak investigations and inform targeted prevention strategies.

We hypothesized that enteric disease outbreaks associated with ruminant contact differ systematically from those associated with non-ruminant animal contact in terms of epidemiologic and clinical characteristics. In this study, we used national outbreak surveillance data to compare the characteristics of enteric illness outbreaks linked to ruminant contact with those linked to non-ruminant animal contact. Our objectives were to (1) characterize differences in outbreak size and etiologic agents, (2) compare patient demographic characteristics, (3) assess differences in healthcare utilization and clinical outcomes, and (4) identify patterns that may guide targeted public health interventions. By explicitly comparing outbreaks across animal contact types, this study aims to address a key gap in the literature and provide evidence to support more tailored prevention and response strategies.

## 2 Methods

### 2.1 Data source

Data for this study were obtained via formal request from the Centers for Disease Control and Prevention (CDC) National Outbreak Reporting System (NORS), specifically the Animal Contact Outbreak Surveillance System (ACOSS) on February 17, 2023. NORS is a national, passive, report-based surveillance system through which state, local, and territorial health departments voluntarily report outbreak data to CDC. ACOSS is the component of NORS that captures enteric disease outbreaks associated with animal contact. Data collected during public health investigations include information on outbreak etiology, transmission mode, implicated animal exposure, outbreak setting, number of illnesses, and geographic scope (single-state or multistate). We obtained all finalized reports of enteric illness outbreaks linked to animal contact that were available for analysis, which included reports as of December 31, 2021. Reportes are reviewed and compiled by CDC; however, completeness and accuracy may vary across jurisdictions because of differences in local investigation and reporting practices.

For this analysis, outbreaks were selected using the following criteria: (1) primary mode of transmission reported as animal contact; (2) laboratory-confirmed etiology of *Campylobacter* spp., *Cryptosporidium* spp., Shiga toxin–producing *Escherichia coli* (STEC), or *Salmonella* spp.; and (3) illness onset date between January 1, 2013, and December 31, 2021. Both single-state and multistate outbreaks were included, as defined by CDC. Outbreaks were excluded if multiple etiologies were reported, if the animal type was unspecified, or if both ruminant and non-ruminant animals were identified as transmission sources.

Animal categories were defined according to CDC classification criteria to ensure consistency. Missing or incomplete data were handled by excluding records with insufficient information for key variables from specific analyses, as applicable. All analytical choices, including inclusion and exclusion criteria and variable definitions, were specified *a priori* to support reproducibility.

As NORS/ACOSS is a passive, report-based surveillance system, the data are subject to limitations including underreporting, potential misclassification, and variability in data quality across jurisdictions. Differences in local investigation capacity and reporting practices may affect the completeness and consistency of the dataset and should be considered when interpreting results.

### 2.2 Definitions and measures

A case was defined as illness in a person linked to an outbreak by the investigating health agency, and outbreaks were defined according to NORS definitions (1). In NORS, each outbreak is assigned a unique CDC identifier (CDCID), and each record represents a single outbreak (which may be single-state or multistate). Single-state outbreaks were defined as outbreaks involving cases from one state or jurisdiction, whereas multistate outbreaks involved cases reported from two or more states or jurisdictions, consistent with CDC NORS definitions. Outbreak size was defined as the estimated total number of primary cases (i.e., both laboratory-confirmed and probable cases) per outbreak, as reported by the investigating agency. Laboratory-confirmed cases had diagnostic evidence of infection, while probable cases were epidemiologically linked to a confirmed case or exposure but lacked diagnostic confirmation. Outbreaks were classified as either ruminant-associated (contact with cattle, sheep, or goats) or non-ruminant-associated (contact with other animal species, excluding ruminants), consistent with CDC categorization schemes. NORS provides aggregated outbreak-level data, meaning that data are summarized at the outbreak level (e.g., total number of cases, distributions of age and sex) rather than individual case-level records for each case.

### 2.3 Statistical analyses

We summarized the number of outbreaks and associated illnesses for each pathogen (*Campylobacter*, *Cryptosporidium*, STEC, and *Salmonella*) by animal contact type (ruminant- vs. non-ruminant-associated) and further described outbreaks by specific animal species within these animal contact categories. Analyses included both single and multistate outbreaks.

Relative frequencies (proportions) were calculated using variable-specific denominators; for each analysis, the denominator included only cases with non-missing data for the variable of interest. Relative frequencies were calculated for sex, age group (0–4, 5–9, 10–19, 20–49, and ≥50 years), healthcare utilization (hospitalization, emergency department visit, or outpatient care), and survival status, both overall and stratified by disease etiology. Accordingly, denominators varied across analyses depending on data completeness.

Outbreak size (number of cases per outbreak) was compared between ruminant- and non-ruminant-associated outbreaks for each etiology using the Wilcoxon rank-sum test, because outbreak size was not normally distributed. Differences in categorical variables (sex, age group, and hospitalization status) between ruminant- and non-ruminant-associated outbreaks were assessed using Pearson’s chi-square test when expected cell counts were adequate and Fisher’s exact test when the expected cell counts were <5.

Analyses were conducted separately by etiology and stratified by animal contact type (ruminant vs. non-ruminant). Single-state and multistate outbreaks were both included in the analyses. Missing data were handled using complete-case analysis for each variable; denominators therefore varied according to the availability of non-missing data for each analysis. All statistical analyses were performed using SAS version 9.4 (SAS Institute Inc., Cary, NC, United States).

## 3 Results

**Figure 1:**
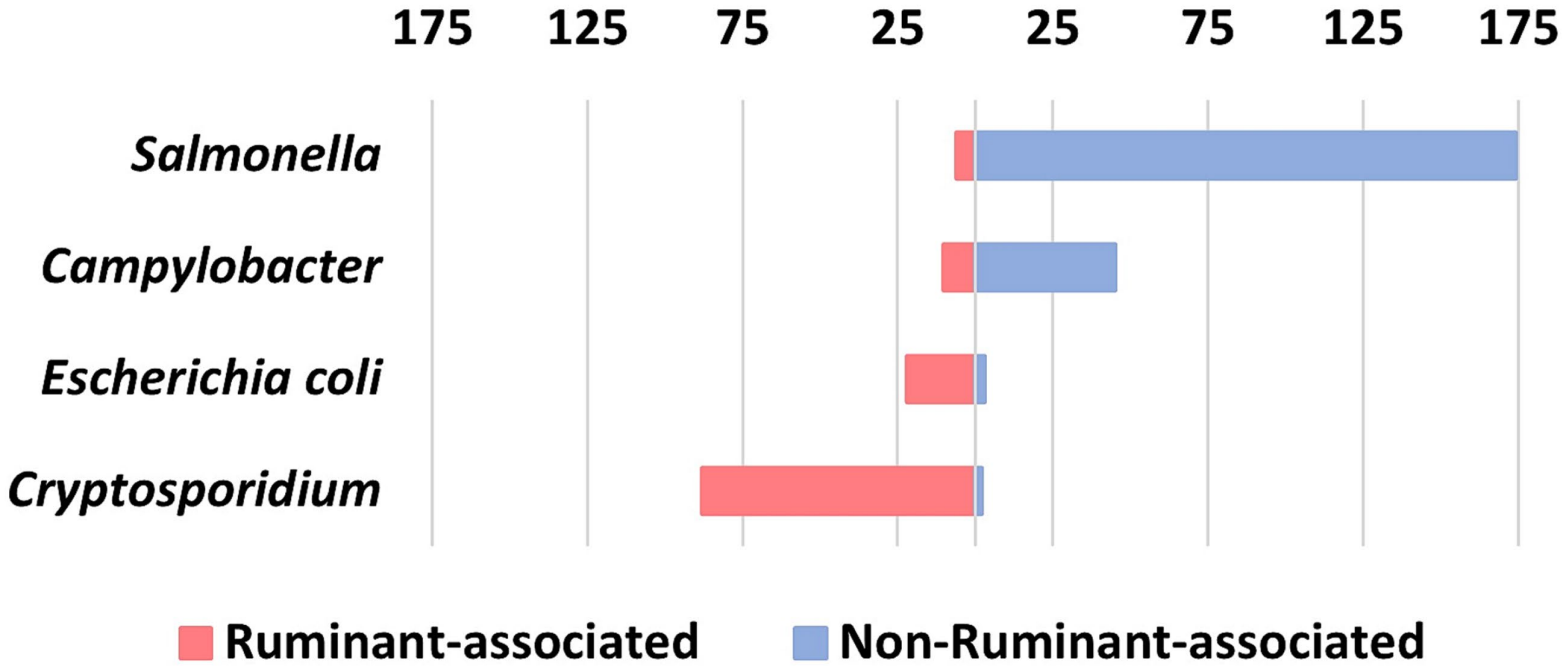
Number of outbreaks associated with ruminant or non-ruminant contact – National Outbreak Reporting System, United States, 2013–2021.

Between 2013 and 2021, a total of 350 outbreaks with animal contact as the primary mode of transmission were reported to NORS and met the inclusion criteria for this study, accounting for 10,741 outbreak-associated illnesses. Of these, 126 outbreaks (36%) involving 1,107 illnesses were linked to ruminant contact, while 224 outbreaks (64%) involving 9,634 illnesses were associated with non-ruminant contact. All ruminant-associated outbreaks occurred within a single state, whereas 120 (54%) of non–ruminant associated outbreaks were multistate and the remaining were single-outbreaks. Overall, *Salmonella* was the most frequently reported outbreak etiology (*n* = 180, 51%), followed by *Cryptosporidium* (*n* = 90, 26%), *Campylobacter* (*n* = 55, 16%), and STEC (*n* = 25, 7%). Most ruminant-associated outbreaks were caused by *Cryptosporidium* (88/126, 70%) and STEC (22/126, 17%), whereas the majority of non-ruminant-associated outbreaks were caused by *Salmonella* (174/224, 78%) and *Campylobacter* (45/224, 20%) (Table 1; Figure 1).

**Table 1:** Characteristics of outbreaks* linked to ruminant or non-ruminant contact by etiology – National Outbreak Reporting System, United States, 2013–2021.

Etiology		All outbreaks *n* = 350 (100%)	Ruminant contact-associated outbreaks *n* = 126 (36%)	Non-ruminant contact-associated outbreaks *n* = 224 (64%)	*p*-value^†^
*Campylobacter*	*n* outbreaks (%)	55 (16%)	10 (8%)	45 (20%)	0.843
	Median Cases (IQR^‡^)	3 (2, 5)	2.5 (2, 6)	3 (2, 4)	
	minimum-maximum	2–120	2–7	2–120	
*Cryptosporidium*	*n* outbreaks (%)	90 (26%)	88 (70%)	2 (1%)	0.456
	Median Cases (IQR^‡^)	4.5 (3, 8)	4.5 (3, 7.5)	9.5 (4, 15)	
	minimum-maximum	2–72	2–72	4–15	
STEC	*n* outbreaks (%)	25 (7%)	22 (17%)	3 (1%)	0.900
	Median Cases (IQR^‡^)	6 (3, 11)	5.5 (3, 11)	7 (3, 11)	
	minimum-maximum	2–205	2–205	3–11	
*Salmonella*	*n* outbreaks (%)	180 (51%)	6 (5%)	174 (78%)	0.001
	Median Cases (IQR^‡^)	19.5 (5, 53.5)	2 (2, 3)	21 (6, 54)	
	minimum-maximum	2–848	2–6	2–848	

^*^All ruminant outbreaks were single-state outbreaks 126 (100%). Among non-ruminant-associated outbreaks, 120/224 (54%) were multistate outbreaks, and the remaining outbreaks were single-state outbreaks.

^†^Wilcoxon rank sum test was used to compare the median number of cases by animal type.

^‡^IQR, Inter-quartile range.

**Figure 2:**
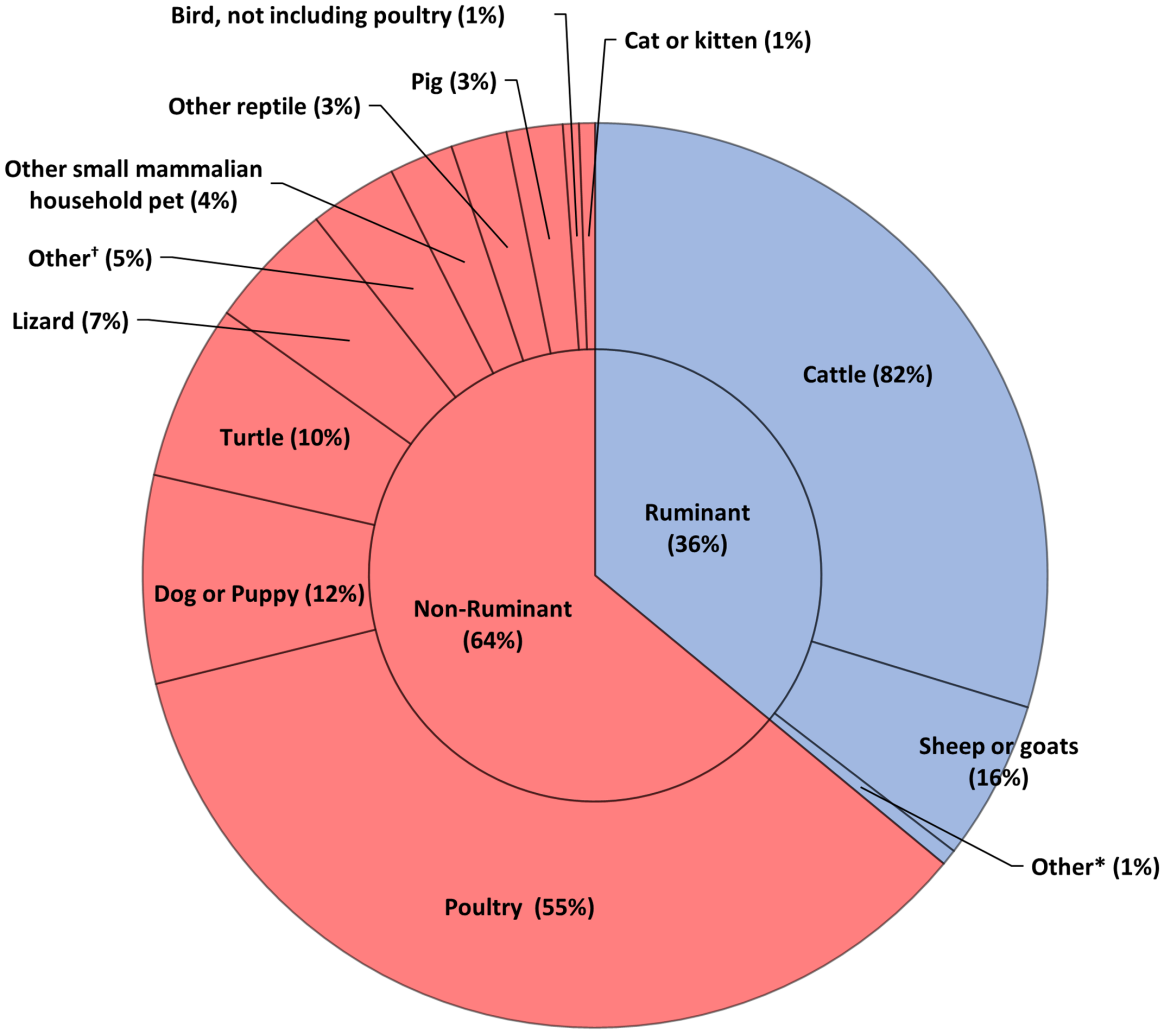
Percentage and count of outbreaks associated with specific ruminants (*n* = 126) and non-ruminants (*n* = 224) – National Outbreak Reporting System, United States, 2013–2021. Footnote: ^*^ “Other” category for ruminant-associated outbreaks was reportedly linked to “cattle, sheep, or goats,” but no further details were provided about the type of animal implicated in this outbreak. ^†^ “Other” category for non-ruminant-associated outbreaks includes outbreaks linked to horses, raccoons, or other livestock. “Small mammal” category includes outbreaks linked to guinea pigs, hedgehogs, mice, or unspecified rodents. “Reptile” category includes outbreaks linked to lizards, bearded dragons, corn snakes, ball pythons, or unspecified reptiles.

Among the 126 ruminant-associated outbreaks, 104 (83%) involved contact with cattle only (Figure 2), most of these outbreaks were caused by *Cryptosporidium* (80/104, 77%). Twenty outbreaks (16%) were linked to sheep or goat contact, including nine (45%) caused by STEC, seven (35%) caused by *Cryptosporidium*, and four (20%) caused by *Campylobacter*. Two outbreaks (2%) involved contact with an unspecified ruminant species and were caused by *Salmonella* and *Cryptosporidium*, respectively.

Of the 224 non-ruminant-associated outbreaks, backyard poultry were the most frequently implicated animals, accounting for 123 outbreaks (55%) (Figure 2). *Salmonella* was the predominant pathogen in poultry-associated outbreaks (108/123, 88%), with *Campylobacter* reported in the remaining 15 outbreaks (13%). Reptiles, including bearded dragons, turtles, ball pythons, and other unspecified species, were the next most common source (45/224, 20%), with 44 outbreaks caused by *Salmonella* and one by *Campylobacter*. Companion animals (e.g., dogs and cats) were linked to 28 outbreaks (28/224, 13%), with nearly all caused by *Campylobacter* (26/28, 93%). Small mammals, such as guinea pigs, rodents (mice and rats), and hedgehogs, were linked to 10 outbreaks (10/224, 4%), all of which were salmonellosis outbreaks.

**Figure 3:**
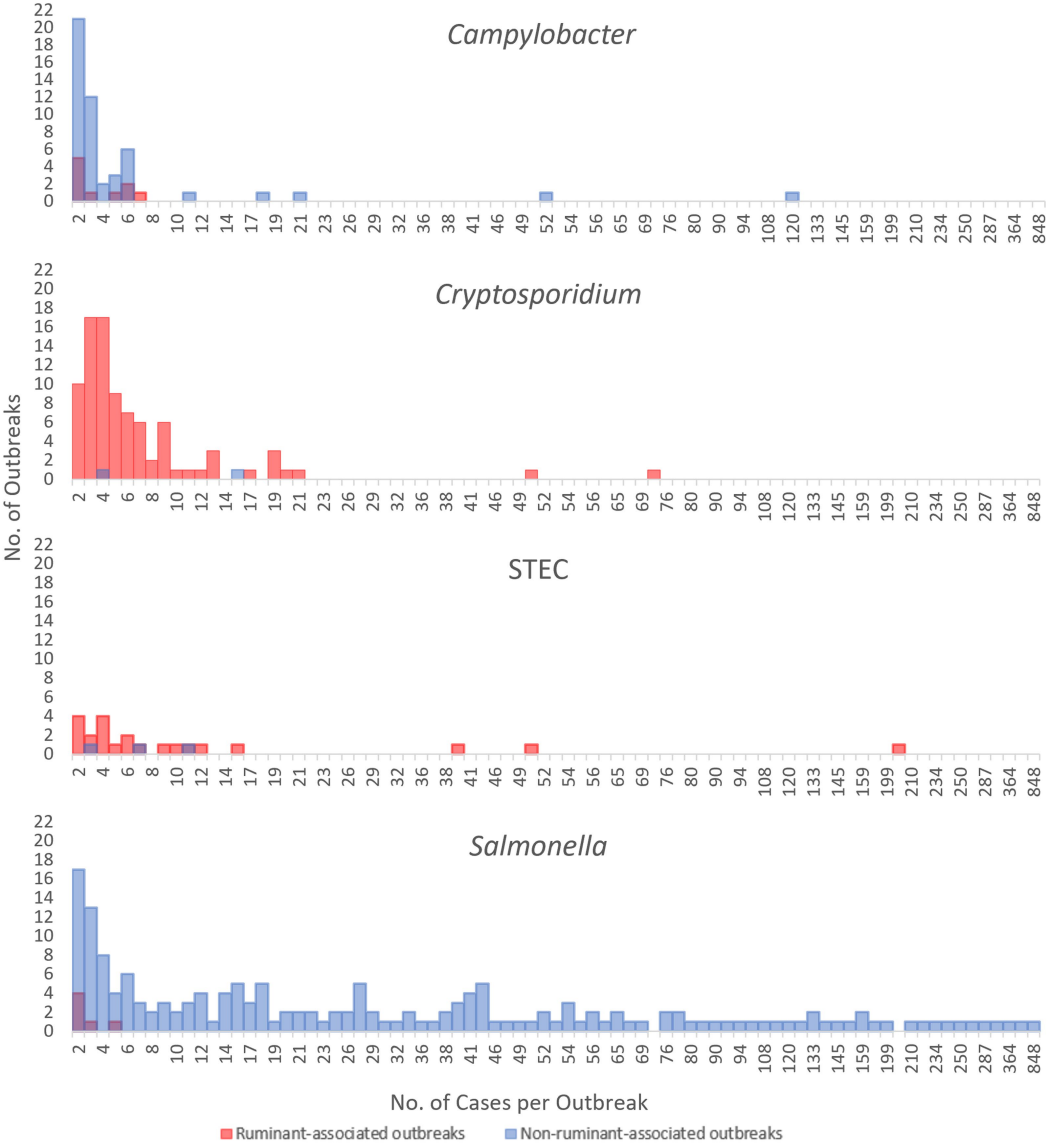
Distribution of outbreak size by pathogen and animal contact type – National Outbreak Reporting System, United States, 2013–2021.

Overall, ruminant-associated outbreaks tended to be smaller in size than non-ruminant-associated outbreaks (median: 4 vs. 14 cases, *p* < 0.001). When stratified by pathogen, outbreak sizes were similar between ruminant-and non-ruminant-associated outbreaks for *Campylobacter*, *Cryptosporidium*, and STEC (Table 1). However, *Salmonella* outbreaks linked to ruminant contact had a smaller median number of cases (2, interquartile range [IQR]: 2–3) compared to non-ruminant-associated *Salmonella* outbreaks (21, IQR: 6–54; *p* = 0.001) (Table 1; Figure 3).

When aggregated across etiologies, 337 (35%) of 954 ruminant-associated outbreak cases with available sex information were male, compared with 4,093 (44%) non-ruminant-associated outbreak cases with available sex information (*p* < 0.001) (Table 2). Ruminant-associated outbreaks had a higher proportion of male cases compared to non-ruminant-associated outbreaks for outbreaks of *Campylobacter* (75% vs. 40%, p < 0.001) and *Cryptosporidium* (36% vs. 11%, *p* = 0.040) (Table 3).

**Table 2:** Comparison of ruminant and non-ruminant outbreak-associated illnesses by sex, age, healthcare utilization, and survival – National Outbreak Reporting System, United States, 2013–2021.

Demographic characteristics and clinical outcomes	All outbreaks	Ruminant contact-associated outbreaks	Non-ruminant contact-associated outbreaks	*p*-value^*^
	*n* cases	*n* cases (%)	*n* cases (%)	
	10,741	1,107 (10.3)	9,634 (89.7)	
Sex				
Male	4,430	337 (35)	4,093 (44)	<0.001
Female	5,761	617 (65)	5,144 (56)	
Unknown	550			
Age group				
0–4 yrs. old	2,446	115 (11)	2,331 (26)	<0.001
5–9 yrs. old	959	228 (22)	731 (8)	
10–19 yrs. old	1,041	164 (16)	877 (10)	
20–49 yrs. old	2,976	446 (43)	2,530 (28)	
≥50 yrs. old	2,715	77 (8)	2,638 (29)	
Unknown	604			
Healthcare utilization				
Hospitalization				
Yes	2,128	92 (11)	2,036 (29)	
No	5,724	761 (89)	4,963 (71)	<0.001
Unknown	2,889			
Emergency room visit				
Yes	175	96 (14)	79 (21)	0.001
No	903	610 (86)	293 (79)	
Unknown	9,663			
Outpatient visit				
Yes	562	331 (44)	231 (50)	0.087
No	650	414 (56)	236 (50)	
Unknown	9,529			
Death				
Yes	13	1 (<1)	12 (<1)	0.999
No	8,255	1,053 (100)	7,202 (100)	
Unknown	2,473			

^*^Chi square was used for comparison when expected cell counts were adequate and Fisher’s exact test when the expected cell counts were <5. Unknowns were not included in statistic test.

**Table 3:** Demographic and hospitalization information for outbreak-associated illnesses linked to ruminant or non-ruminant contact, by etiology – National Outbreak Reporting System, United States, 2013–2021.

Demographic characteristics and clinical outcomes	All outbreaks	Ruminant contact-associated outbreaks	Non-ruminant contact-associated outbreaks	*p*-value^*^
	*n* cases (%)	*n* cases (%)	*n* cases (%)	
*Campylobacter*	372	37	335	
Sex				<0.001
Male	153 (43)	24 (75)	129 (40)	
Female	202 (57)	8 (25)	194 (60)	
Unknown	17	5	12	
Age groups				0.341
0–4 yrs. old	31 (9)	2 (6)	29 (9)	
5–9 yrs. old	24 (7)	2 (6)	22 (7)	
10–19 yrs. old	57 (16)	3 (9)	54 (17)	
20–49 yrs. old	171 (48)	21 (66)	150 (46)	
≥50 yrs. old	72 (20)	4 (13)	68 (21)	
Unknown	17	5	12	
Hospitalization				0.065
Yes	40 (12)	1 (3)	39 (13)	
No	292 (88)	36 (97)	256 (87)	
Unknown	40	0	40	
*Cryptosporidium*	666	647	19	
Sex				0.04
Male	224 (35)	222 (36)	2 (11)	
Female	412 (65)	397 (64)	15 (79)	
Unknown	30	28	2	
Age groups				0.182
0–4 yrs. old	43 (7)	43 (7)	0 (0)	
5–9 yrs. old	118 (20)	118 (20)	0 (0)	
10–19 yrs. old	115 (19)	111 (19)	4 (29)	
20–49 yrs. old	299 (50)	289 (49)	10 (71)	
≥50 yrs. old	26 (4)	26 (4)	0 (0)	
Unknown	65	60	5	
Hospitalization				0.616
Yes	34 (5)	34 (6)	0 (0)	
No	592 (95)	574 (94)	18 (100)	
Unknown	40	39	1	
STEC	427	406	21	
Sex				0.292
Male	83 (28)	82 (29)	1 (10)	
Female	213 (72)	204 (71)	9 (90)	
Unknown	131	120	11	
Age groups				0.009
0–4 yrs. old	69 (17)	66 (17)	3 (30)	
5–9 yrs. old	105 (26)	104 (26)	1 (10)	
10–19 yrs. old	55 (14)	50 (13)	5 (50)	
20–49 yrs. old	132 (33)	131 (33)	1 (10)	
≥50 yrs. old	43 (11)	43 (11)	0 (0)	
Unknown	23	12	11	
Hospitalization				0.474
Yes	57 (28)	53 (28)	4 (40)	
No	144 (72)	138 (72)	6 (60)	
Unknown	226	215	11	
*Salmonella*	9,276	17	9,259	
Sex				0.488
Male	3,970 (45)	9 (53)	3,961 (45)	
Female	4,934 (55)	8 (47)	4,926 (55)	
Unknown	372	0	372	
Age group				0.195
0–4 yrs. old	2,303 (26)	4 (24)	2,299 (26)	
5–9 yrs. old	712 (8)	4 (24)	708 (8)	
10–19 yrs. old	814 (9)	0 (0)	814 (9)	
20–49 yrs. old	2,374 (27)	5 (29)	2,369 (27)	
≥50 yrs. old	2,574 (29)	4 (24)	2,570 (29)	
Unknown	499	0	499	
Hospitalization				0.791
Yes	1997 (30)	4 (24)	1993 (30)	
No	4,696 (70)	13 (76)	4,683 (70)	
Unknown	2,583	0	2,583	

^*^Chi square was used for comparison when expected cell counts were adequate and Fisher’s exact test when the expected cell counts were <5. Unknowns were not included in statistic test.

Ruminant-associated outbreaks had higher proportions of cases aged 5–9 years (22% vs. 8%), 10–19 years (16% vs. 10%), and 20–49 years (43% vs. 28%), while non-ruminant-associated outbreaks had a greater proportion of cases aged 0–4 years (26% vs. 11%) and ≥50 years (29% vs. 8%) (Table 2). When comparing the age distribution of ruminant- and non-ruminant outbreaks for each of the pathogens, significant differences were only observed for outbreaks caused by STEC (*p* = 0.009) (Table 3).

Data on hospitalization, emergency room (ER) visits, and outpatient care were available for 7,852, 1,078, and 1,212 case-patients, respectively (Table 2). Ruminant-associated outbreaks had significantly lower proportions of hospitalized cases than non-ruminant-associated outbreaks (11% vs. 29%, *p* < 0.001) and ER visits (14% vs. 21%, *p* = 0.001), but no significant difference was observed in outpatient care visits (*p* = 0.087). One death was reported in ruminant-associated outbreaks, compared to 12 deaths in non-ruminant-associated outbreaks. When stratified by etiology, there were no significant differences between ruminant- and non-ruminant–associated outbreaks in hospitalization (Table 3), in emergency department visits, or outpatient care (results not shown) (all *p* > 0.05), with the exception of *Salmonella*, for which ruminant-associated outbreaks had a significantly higher proportion of cases seeking outpatient care (76% vs. 48%, *p* = 0.020). Death counts were too low for statistical comparison.

## 4 Discussion

Our analysis of 350 enteric disease outbreaks associated with animal contact revealed distinct epidemiologic patterns that varied by the type of animal exposure. Although more outbreaks were linked to contact with non-ruminants, this category represents a more diverse group of animals including backyard poultry, reptiles, small mammals, companion animals, and non-ruminant livestock (e.g., pigs and poultry). In contrast, ruminant-associated outbreaks were only linked to cattle, goats, or sheep, with cattle the most common source of these outbreaks. An analysis of ACOSS data similarly found that cattle, backyard poultry, and turtles represent the most important animal sources for enteric illness outbreaks in the United States, making them important targets for public health interventions (5). In this analysis, we used ruminant versus non-ruminant animal contact as the primary framework for interpreting differences in outbreak epidemiology across pathogens, demographics, and outcomes.

Within this framework, pathogen distributions differed systematically by animal exposure type. Ruminant-associated outbreaks were more commonly caused by *Cryptosporidium* or STEC, whereas non-ruminant-associated outbreaks were more commonly caused by *Salmonella* or *Campylobacter*. Prior studies have demonstrated that ruminants, particularly cattle, are important reservoirs for *Cryptosporidium* and can shed large quantities of oocysts into the environment, facilitating transmission in public settings such as farms and petting zoos (9, 18). Additionally, previous analyses of surveillance data and outbreak reports have characterized animal species implicated as sources of outbreaks by pathogen type (8–12, 15–17). Our findings extend this work by demonstrating clear differences between ruminant- and non-ruminant-associated outbreaks. During epidemiologic investigations of outbreaks associated with venues where the public can contact multiple animal types (e.g., petting zoos, agricultural fairs, or farms), these data may support hypothesis generation and decision-making regarding which animal types to prioritize for testing.

Despite these differences in pathogen distribution between ruminant and non-ruminant exposures, outbreaks of all four pathogens included in this analysis were represented in both ruminant and non-ruminant categories. Despite the diversity of enteric pathogens that can be transmitted from animals to people, prevention of illness caused by each of these pathogens relies on similar principles such as adequate hand hygiene, environmental biosecurity, proper animal husbandry, and safe animal handling practices (17, 19). While illness prevention strategies are nuanced based on the type of animal and the setting in which the animal is kept, these strategies largely will not differ based on the type of pathogen implicated in an outbreak.

Age distributions also varied by animal exposure type, with ruminant-associated outbreaks more commonly affecting individuals aged 5–49 years, potentially reflecting exposures related to occupational, agricultural, or educational activities. A Finnish case–control study demonstrated an association between cryptosporidiosis and cattle exposure among individuals engaged in agricultural work or studies (20). Conversely, non-ruminant-associated outbreaks more commonly affected children aged 0–4 and adults aged ≥50. Outbreaks linked to non-ruminants may more closely reflect household pet ownership. These age-related trends can be helpful for identifying groups that can share and amplify public health messaging to the public. For example, pet retailers and veterinarians who care for household pets may help disseminate information about preventing enteric illnesses, particularly to households with children under the age of 5 or with older adults.

Outbreak size and severity further distinguished ruminant and non-ruminant exposures. Ruminant-associated outbreaks were typically smaller in size than non-ruminant-associated outbreaks and resulted in fewer hospitalizations and deaths. The smaller outbreak sizes associated with ruminant exposure may reflect more localized transmission settings, such as farms or agricultural education programs. This finding is also likely impacted by the recurrent public health challenge of backyard poultry-associated salmonellosis. Backyard poultry ownership has grown steadily in the United States, and as a result, large multistate outbreaks occur each year linked to contact with poultry (14, 16). Backyard poultry are distributed across the country and shed *Salmonella* into the environment even when appearing healthy and clean. No other type of live animal contributes as significantly to human enteric illness outbreaks (5, 14, 16).

Important limitations of this analysis should be considered. Although outbreak-level data provide valuable insights into broad epidemiological patterns, the lack of individual-level information, such as specific animal handling and husbandry practices, duration of exposure, and other patient-reported details limits our ability to characterize differences between these types of outbreaks. Additionally, there is potential for underreporting and misclassification of outbreaks, particularly in informal settings such as backyard farms, urban agriculture, and exotic pet ownership, which may bias observed patterns. It is possible that not all outbreaks linked to animal contact during the study period were reported to NORS. Variation in reporting practices and public health capacity across jurisdictions may further affect the completeness and consistency of the data.

An additional limitation is that all ruminant-associated outbreaks identified in this dataset were single-state outbreaks, whereas a substantial proportion of non-ruminant outbreaks were multistate. Multistate outbreaks may differ from single-state outbreaks in several ways, including outbreak detection, investigation intensity, traceback capacity, case ascertainment, and geographic distribution of exposure. Multistate outbreaks are also more likely to involve commercially distributed animals or products and may therefore be identified and investigated differently than localized outbreaks. As a result, direct comparisons between ruminant- and non-ruminant-associated outbreaks should be interpreted cautiously because some observed differences may reflect differences in outbreak structure and surveillance practices rather than true epidemiologic differences.

Finally, data after 2021 were not included because finalized outbreak reports for subsequent years were not yet fully available in NORS/ACOSS at the time of data extraction and analysis. Reporting and data finalization delays may limit the completeness of more recent surveillance data and could affect interpretation of temporal trends. Continued evaluation using updated NORS data will be valuable for assessing whether the epidemiologic patterns identified in this study persist in subsequent years.

## 5 Conclusion

Our findings highlight the importance of tailored, animal-specific risk communication in preventing zoonotic disease transmission and support a One Health perspective recognizing the interconnected roles of humans, animals, and the environment in enteric pathogen transmission (2, 20). Framing these findings by animal exposure type also helps translate epidemiologic patterns into actionable public health interventions. Ruminant-associated outbreaks affected working-age adults most commonly, which could represent exposures in farming or agricultural settings, emphasizing the importance of targeted hygiene education and protective measures for this population. In contrast, non-ruminant-associated outbreaks—particularly those linked to household pets and reptiles—may require broader public health messaging on safe handling practices across all age groups. This is especially important for households with young children and older adults, who are more vulnerable to severe illness. Overall, characterizing outbreaks by animal contact type remains a critical tool for public health surveillance and response, providing a practical foundation for anticipating risks, designing focused interventions, and guiding future epidemiologic investigations of zoonotic enteric diseases.

## Data Availability

Publicly available datasets were analyzed in this study. These data can be requested from NORS: https://www.cdc.gov/nors/data/index.html.
